# Lockdown Stories: A Qualitative Assessment and Comprehensive Taxonomy of Career Resources

**DOI:** 10.1177/10690727241287533

**Published:** 2024-09-24

**Authors:** Shékina Rochat, Caroline Arnoux-Nicolas, William A. Borgen

**Affiliations:** 1 27213 University of Lausanne, Lausanne, Switzerland; 2 27044 University of Paris-Nanterre, Nanterre, France; 3 8166 University of British Columbia, Vancouver, BC, Canada

**Keywords:** taxonomy, career resources, career obstacles, COVID-19, narrative

## Abstract

Career resources are receiving increasing attention in the context of career development. This paper utilizes M. E. Ford’s (1992) ten components of effective functioning to provide a comprehensive typology of factors likely to act as career resources and test this proposition in a context of career shock with a narrative design. In the weeks following the first COVID-19 lockdown, 42 participants were asked to complete a questionnaire about their well-being, perceived employability, and emotional anticipation of their career future, as well as to write three stories about their experience with the lockdown. M. E. Ford’s categories were used to identify and code the resources and obstacles mentioned in the stories. Results show the relevance of such a taxonomy to classify both career resources and obstacles. Additionally, the type of story (general story, positive or negative story) in which career resources and obstacles were mentioned played a significant role in their association with the quantitative measures. Conceptual and practical implications are discussed.

Due to globalization, the rise of new technologies, global warming and accelerating rhythms, today’s world of work is characterized by increasing instability and uncertainty, making career transitions more frequent and challenging (Akkermans et al., 2024). In particular, career paths are all the more likely to be impacted by *career shocks* which refer to “disruptive and extraordinary events that are, at least to some degree, caused by factors outside the focal individuals’ control and that trigger a deliberate thought process concerning one’s career” (Akkermans et al., 2018, p. 4). Empirical research established that such career shocks have a substantial impact on various career outcomes such as career success (Blokker et al., 2019), career optimism (Hofer et al., 2021), work motivation (Pak et al., 2021), and career decision-making (Rummel et al., 2021). However, key challenges remained to deepen the understanding of career shocks, including how they impact individuals’ career development (Akkermans et al., 2021). To this end, Akkermans et al. (2018) encouraged the use of qualitative approaches. Recently, Akkermans et al. (2020) suggested that such an impact will depend on the accessibility and utilization of *career resources*. In fact, resources play a central role in ensuring sustainable continuity and well-being in career paths, despite career shocks (De Vos et al., 2020).

To properly understand the role of career resources regarding career shocks, it is essential to clarify first *what* these resources are, which is still considered a significant challenge (Hobfoll et al., 2018). Early on, (Hobfoll, 1989) defined resources as “those objects, personal characteristics, conditions, or energies that are valued by individuals or that serve as a means for the attainment of these objects, personal characteristics, conditions or energies” (p. 516). This definition was later enlarged to include “anything perceived by the individual to help attain his or her goals” (Halbesleben et al., 2014, p. 1338). To adequately assess resources despite such a broad definition, Halbesleben et al. (2014) notably encouraged researchers to rely on the categories suggested in resources taxonomies. In this article, we propose that M. E. Ford’s (1992) model of effective functioning offers an excellent meta-theory to categorize and conceptualize factors that may act as career resources or obstacles, in a complete way.

To support this assumption, we will introduce this model and use it to assess the resources and obstacles contained in short stories of working adults about their experience with the first COVID-19 lockdown. In fact, the COVID-19 pandemic can be understood as a “career shock” which disrupted individuals career paths and routine in an extraordinary way (Akkermans et al., 2020), and thus required individuals to mobilize their career resources (Borgen & Borgen, 2022). Pak et al. (2021) already shown that qualitative approaches help understand the mechanisms of career resources after a career shock. Additionally, McMahon et al. (2012) demonstrated that resources can be qualitatively investigated through the stories of career transition and adaptability. Accordingly, we aim to extend the knowledge on career resources in three different ways: (1) proposing an exhaustive taxonomy of resources anchored on M. E. Ford’s (1992) model of effective functioning; (2) using a narrative approach to investigate the relevance of Ford’s (1992) categories to account for factors that may act as career resources or obstacles in an exhaustive yet concise way; (3) examining the relationships between those resources and obstacles with indicators of well-being, perceived employability, and emotional anticipation of the career future.

Several authors have proposed theoretical models and taxonomies of career resources that can contribute to career success (i.e., the Career Resources Model; Hirschi, 2012; Hirschi et al., 2018) or well-being and performance at work (i.e., the PERMA+4 Model; Donaldson et al., 2022; Donaldson & Donaldson, 2020). However, neither of these models is anchored on a sound theoretical framework or a broader meta-theory of individuals’ behavior and development which would guide the selection of relevant components, as well as shed light on their relationships (Donaldson et al., 2022). Additionally, while it is impossible (and undesirable) to list all the possible career resources (Hirschi et al., 2018), important types of resources seem to be missing from both, as each model contains categories that the other does not have.

These two models also rely on the key assumption that certain factors (e.g., being skilled, optimistic and having social support) are positive in themselves across situations, and thus constitute desirable resources that should be promoted to foster individuals’ optimal functioning (McNulty & Fincham, 2012). However, “Hobfoll (1988, 1998) clarified [that] how resources operated depended on the ecological context, such that in one context a resource might be salient and positive and in another, might be salient but negative” (Hobfoll et al., 2018, p. 113). This claim is supported by the growing evidence which shows that traits (e.g., self-esteem; Neff & Vonk, 2009), conditions (e.g., social support; Beehr et al., 2010) processes (e.g., regulatory strategies; Bonanno & Burton, 2013), and outcomes (e.g., career success; Spurk et al., 2019) that are usually considered as “positive” might backfire under certain situations. Accordingly, a comprehensive model of career resources should be able to account for the fact that a given factor might act as a resource or a barrier for career success, performance, and well-being, depending on individuals’ set of circumstances.

## Theoretical Background and Hypothesis Development

The above literature review highlighted the lack of a theoretically grounded, comprehensive, balanced, contextual, and adequately measured model of career resources. Therefore, we propose to adopt M. E. Ford’s (1992) far-reaching model of the human functioning. This model postulates that individuals’ ability to attain their goals at a given time depends on ten factors grouped in four main categories: (1) motivation (i.e., activated goals, personal beliefs, contextual beliefs, and emotions), (2) skills, (3) biological functioning; and (4) environment (i.e., natural, material, social, and socio-cultural contexts). The model is anchored in the Living Systems Framework (LSF; D. H. Ford, 1987): a comprehensive, integrative, and evidence-based theory of individuals’ behavior and development from their conception to their death. The LSF is increasingly considered as an invaluable way to foster understanding in human science (M. E. Ford & Smith, 2020), and was recently adapted to the context of vocational psychology (see Vondracek et al., 2014, 2020).

In M. E. Ford’s (1992) model, motivation is defined as the structural pattern of: (1) goals, which represent the states a person wish to achieve or avoid; (2) personal agency beliefs—consisting of (a) personal belief (i.e., the perceived personal skills, or self-efficacy beliefs), and (b) context beliefs (i.e., the perceived responsiveness of the context), which provide information about the attainability a considered goal; and (3) emotions, that inform about the goal *desirability,* and energize subsequent actions. Effective functioning will also depend on the person’s actual skills and responsive environment, beyond their mere perception. Skills denote cognitive knowledge, physical action capabilities (i.e., “know how”), as well as soft skills (e.g., personality traits and enduring interests) pertaining to the selection and attainment of goals. Motivation and skills are closely dependent upon various biological systems (e.g., health, stress, and biological capabilities), including neurological functioning and basic functions (e.g., nutrition, elimination, sleep, exercise, and so on).

In M. E. Ford’s (1992) model, the environment is considered to be an integral part of the individual functioning, and is posited to encompass (1) natural context (i.e., products of nature, such as meteorological events, vegetal or mineral elements, etc.), (2) material context (i.e., physical and symbolic products of humans, such as money, infrastructure, pieces of art, etc.), (3) social context (i.e., close or distant people, such as family members, colleagues, role models, etc.), and (4) socio-cultural context (i.e., influential institutions and traditions, such as educational systems, languages, religion, etc.).

As illustrated in Table 1, M. E. Ford’s (1992) model of effective functioning encompasses all the categories of resources previously identified in the Career Resources Model (Hirschi, 2012; Hirschi et al., 2018) and the PERMA+4 (Donaldson et al., 2022; Donaldson & Donaldson, 2020). Accordingly, it allows for gathering and capitalizing on peoples’ strengths. Additionally, the model highlights that all these components are intrinsically interdependent, implying that if one of the dimensions is flawed, it will compromise the effective functioning of the individual. In recent developments of the model (M. E. Ford & Smith, 2007, 2020), the authors also specified that the most important feature for effective functioning is that the components should be *equipoised*, that is balanced and adapted to the context. Hence, this model explicitly acknowledges the fact that each factor can potentially act as a resource or an obstacle in each situation and goal pursuit (Rochat et al., 2017).

**Table 1.** Comparisons between M. E. Ford’s (1992) Model of Effective Functioning with Hirschi et al.’s (2018) Career resources Model and Donaldson and Donaldson’s (2020) PERMA+4 Model of Well-Being at Work.

Effective Functioning	Career Resources Model	PERMA+4
Motivation		
Goals	Career clarity	Meaning (work worth, transcendence, and direction)
		Accomplishment (goals)
Personal beliefs	Career confidence	Mindset (self-efficacy, hope, resilience, grit, growth mindset)
Context beliefs	Ø	Mindset (optimism)
Emotions	Career involvement	Positive emotions (e.g., joy, enthusiasm)
Competences		
Skills	Human capital (occupational expertise, job market knowledge, soft skills)	ø
	Career management behaviors (networking, career exploration, learning)	
Biology		
Biological functioning	Ø	Health (biological, functional, and psychological)
Context		
Natural context	Ø	Physical environment
Material context	Ø	Physical environment, Economic security (income, medical spending, financial saving)
Social context	Social career support	Relationships (giving and perceived), psychosocial environment
Socio-cultural context	Career opportunities, organizational support, job challenge	Economic security (job security)

### A Comprehensive Taxonomy of Career Resources

Based on these considerations, we propose to consider that M. E. Ford’s (1992) model of effective functioning represents a relevant integrative, comprehensive, balanced, contextual and theoretically anchored model for establishing a taxonomy of factors that might act as resources for career success, performance, and well-being. More precisely, we propose that all the individuals’ resources mobilized during a career shock such as the COVID-19 lockdown could be organized into the ten categories of factors listed in the model (i.e., goals, personal beliefs, contextual beliefs, emotions, skills biological functioning, natural context, material context, social context, and socio-cultural context). Given that resources’ value strongly depends on context (Hobfoll et al., 2018), and relies on the concept of *equipoise* (M. E. Ford & Smith, 2007, 2020), we further hypothesize that the same categories could account for both the factors associated with flourishing and those associated with obstacles.

Hypothesis 1 *Career resources and obstacles in response to a career shock can be comprehensively classified in the ten categories of M. E.* Ford’s (1992) *model of effective functioning.*

### The Importance of the Type of Story Narrated

The above literature review emphasized the need for a balanced and contextual understanding of factors likely to act as career resources or obstacles, which implies exploring the circumstance of their occurrence to account of their effect. Here, we posit that a relevant way to investigate it is to consider the type of narrative in which they are described. In fact, McMahon et al. (2012) indicated that resources can be identified through career narratives. Additionally, several researchers (e.g., Bauer et al., 2019; McAdams et al., 2001) have shown that different types of life stories narrative scenes (e.g., that of a turning point, a low point, and a high point) contained distinctive explanation styles (e.g., contamination and redemption), which in turn were differentially associated with self-reported measures of well-being. Accordingly, we posit that different types of stories (i.e., general account of the lockdown experience, high point or low point experienced during the lockdown) contain different types of resources and obstacles, which are then distinctively associated with- and predict individuals’ well-being, perceived employability, and emotional anticipation of their future career following the COVID-19 career shock.

Hypothesis 2 *Different types of stories contain different types of resources and obstacles, and hold an incremental value to the relationship between career resources and obstacles and the level of well-being, perceived employability, and emotional anticipation of the career future*.

## Method

### Procedure

Participants were recruited through social networks and asked to fill out an online survey on “Career resources and well-being during COVID-19.” Eligibility criteria included being more than 18 years of age, working, and being fluent in French and/or English. The data were collected between June 15 and July 6th 2020, that is a month and a half after the end of the first lockdown in Canada and Europe. The questionnaire was available both in English and in French. After providing demographic information about their current situation (i.e., age, sex, country, marital status, number of children in the household, residency type, employment status, type of contract, field of occupation, and COVID-19 impact on working arrangements), participants were asked to write three short narratives about their experiences during the lockdown. They also were asked to complete four short scales assessing well-being, employability, and emotional anticipation of the career future. This study has been approved by the Behavior Research Ethics Board of the University of British Columbia (number H20-1096).

### Participants

Forty-two participants (11.9% male) completed the survey. 75.00% took the survey in French, and 25.00% in English. 72.7% of the participants originated from Switzerland, 15.9% from Canada, and 11.3% from different European Countries (France = 4.76%, Italy = 2.38%, and Portugal = 2.38%). Participants were 41.64 years old on average (*SD* = 10.53). Table 2 details participants’ further sociodemographic information related to their personal and working conditions.

**Table 2.** Participants’ Sociodemographic Information (*N* = 42).

	*n*	(%)
Ethnicity		
White	37	(81.10%)
Asian/Pacific Islander	1	(2.38%)
Hispanic or Latino	1	(2.38%)
Other	3	(7.14%)
Marital status		
Single (never married)	11	(26.19%)
Married	15	(35.71%)
In a domestic partnership	8	(19.05%)
Divorced	7	(16.67%)
Widowed	1	(2.38%)
Children in household		
0	27	(64.29%)
1	7	(16.67%)
2	6	(14.29%)
3	2	(4.76%)
Residence type		
Rental apartment	24	(57.14%)
House or condominium	18	(42.86%)
Employment status		
Employed full-time	35	(83.33%)
Employed part-time	4	(9.52%)
Unemployed	1	(2.38%)
Self-employed	2	(4.76%)
Type of working contract		
Permanent contract	33	(78.57%)
Temporary contract	5	(11.90%)
Casual contract	1	(2.38%)
Other	3	(7.14%)
Field of occupation		
Food, family economy	1	(2.38%)
Economy administration	3	(7.14%)
Electricity, electronics	1	(2.38%)
Teaching	6	(14.29%)
Mechanics, watchmaking, metallurgy	2	(4.76%)
Medicine, health	6	(14.29%)
Social, human sciences	21	(50.00%
Sale purchase	1	(2.38%)
Other	1	(2.38%)
COVID-19’s impact on work		
Now work remotely	31	(73.81%)
Still work as usual	7	(16.67%)
Now work remotely and as usual	2	(4.76%)
Other	2	(4.76%)

### Instruments

#### Short Stories

Following Bauer et al. (2019) use of narrative prompts, participants were asked to write three short stories about their COVID-19 lockdown experience: (1) the story of their life during the lockdown from the beginning until now (i.e., General Story); (2) a high point (i.e., Positive Story); and (3) a low point (i.e., Negative Story) they experienced during the lockdown. The prompt for the General Story was the following: “Could you write, in a few lines, the story of your life during the lockdown from the beginning until now?” Instruction for the Positive and Negative Stories was the following: “Below you will find space to write a short narrative about a *high* point (respectively *low point*) you experienced during the COVID-19 lockdown.” High points were described as experiences of a great uplifting, joy, excitement, contentment, or some other highly positive emotional experiences. Low points were described as experiences of ‘negative’ emotions, such as despair, disillusionment, anxiety, guilt, sadness, etc. For the Positive and Negative Stories, participants were asked to specify what the event was, when and where it happened, who was there; what they were thinking and feeling, and why the event was significant to them.

#### Well-Being

We used Diener et al.’ (2010) Flourishing Scale to measure participants’ well-being. The scale consists of eight items which assess participants’ perceptions of meaning in life, mutually supportive and respectful relationships, engagement in daily activities, sense of competences, optimism, and overall goodness of life. These are rated on a 7-point Likert-scale ranging from 1 “Strongly disagree” to 7 “Strongly agree.” The reliability of this scale was found to be good, with a Cronbach’s alpha of .87 (Diener et al., 2010). For the French version, we used Villieux et al.’s (2016) translation which showed good reliability (α = .82), which is similar to that found in the present study (α = .81).

#### Perceived Employability

Perceived employability was assessed with Berntson and Marklund’s (2007) five item index which assessed participants perceived competencies, network, knowledge of organizations/companies, personal qualities, and experience. The items were rated on a 5-point Likert-scale ranging from 1 “Strongly disagree” to 5 “Strongly agree.” The reliability of this scale was found to be good, with a Cronbach’s alpha of .88 (Berntson & Marklund, 2007). For the French version, the first authors of this article and an experienced career counselor translated the items independently, and then compared their versions and sought an agreement for the best version. This translation showed good reliability (α = .82) in the present study.

#### Emotional Anticipations

The Positive and Negative Affect Scale (PANAS; Watson et al., 1988) was used to assess the participants emotional anticipation of their career future. The scale comprises 20 items in total, ten of which assess positive emotional states (PANAS+) and ten negative emotional states (PANAS-). These items are rated on a 5-point Likert-scale ranging from 1 “Very slightly or not at all” to 5 “Extremely.” The reliability of these scales was found to be good, with a Cronbach’s alpha ranging between .86 and .90 for the positive affect subscale, and between .85 to .87 for the negative affect subscale (Watson et al., 1988). For the French version, we used Bouffard and Lapierre’s (1997) translation which demonstrated excellent reliability for the positive affect subscale (α = .90), and fair for the negative affect subscale (α = .77). For this study, the instruction asked participants to what extent they feel this way when they think of their career future (see Parmentier et al., 2021, for a similar adaptation). The subscales showed good reliability for both the positive (α = .89) and the negative affect (α = .87).

### Analysis

#### Coding Process

Content from the three short stories were fully copy-pasted on Word and Excel. The first author divided the transcripts into units of analysis (i.e., statements; Milne & Adler, 1999), and then the first and second authors independently coded these statements based on the M. E. Ford’s (1992) ten categories of effective functioning: goals (GOAL), personal beliefs (PEB), contextual beliefs (COB), emotions (EMO), skills (SKILL), biological functioning (BIO); natural context (NAT), material context (MAT), social context (SOC), and socio-cultural context (SCU). These factors were also classified according to their mention as resources (e.g., GOAL+) or as obstacles (e.g., GOAL-). Eventually, a neutral category (NEUTRAL) was added to account for the statements that could not be identified either as resources or as obstacles within the context of the sentence or story (the detailed coding table is available from the first author upon request). Both coders are Caucasian female (visiting) Assistant Professors and researchers in the field of vocational psychology. After coding the sessions, the inter-agreement reliability was calculated.

#### Inter-Rater Reliability

Cohen’s kappa was used to assess the inter-rater agreement reliability for counselor’s behaviors and client’s talk throughout the three sessions. Six cases (111 statements) were used as training material, while the 38 remaining cases (771 statements) were double coded to compute the inter-rater agreement reliability. Seven statements were coded without valence from one or other of the coders, making it impossible to include them in the inter-rater reliability computation. In line with Landis and Koch (1977) benchmarks, inter-rater reliability was excellent (K = .81) for the overall coding of the 764 remaining statements.

#### Statistical Analysis

Statistical analyses were performed on Jamovi (1.6.23.0). Differences between the three different types of stories and quantitative measures were computed using Friedman’s nonparametric ANOVA. Spearman’s coefficient correlations were used to assess the relationship between the quantitative data and the types of resources and obstacles, as well as with the number of resources and obstacles mentioned within the three different types of short stories (general story, positive story, and negative story). Hierarchical regressions were used to assess the incremental value of these three types of stories.

## Results

### Comprehensiveness of M. E. Ford’s (1992) Taxonomy

Table 3 displays examples and the number of occurrences of each resource and obstacles narrated by the participants across the three types of stories (i.e., General Story, Positive Story, and Negative Story). 94.16% of the statements were successfully classified into M. E. Ford’s (1992) categories as resources or obstacles, with only 3.30% of the statements, produced by 16 participants, which were coded as “neutral”—that is neither identifiable as a resource nor an obstacle. However, 22 statements (2.54%), produced by 14 participants, could not be classified in any categories of M. E. Ford’s (1992) model, as they were related to the lack of or the disposal of *time*. Therefore, we added a supplementary category (TIME) to account for these statements (with having time as a resource, and lacking time as an obstacle). Therefore, Hypothesis 1 is partially validated.

**Table 3.** Examples and Frequencies of Resources and Obstacles Coded after M. E. Ford’s (1992) Ten Components of Effective Functioning.

Resources				Obstacles			
Code	Freq	*n*	Examples	Code	Freq	*n*	Example
GOAL+	21	17	I Immediately found meaning in my work again (P002)	GOAL-	10	4	I was having trouble finding meaning (P035)
PEB+	10	8	I felt well positioned to work from home (P010)	PEB-	14	11	[I] felt like I couldn’t do either one well (P001)
COB+	25	15	[It allowed me] to maintain a positive vision of the future (P033)	COB-	47	21	Things are still so uncertain (P005)
EMO+	70	29	A lot of pleasure (P015)	EMO-	108	40	I worried (P013)
SKILL+	77	30	[It] allowed me to see the situation from a new perspective (P037)	SKILL-	14	12	My ability to cope is challenged (P036)
BIO+	43	24	Lucky to be healthy (P012)	BIO-	37	17	I had difficulty breathing (P014)
NAT+	20	11	[It allowed me] to enjoy the great outdoors (P021)	NAT-	0		-
MAT+	23	14	The comfort of my apartment [makes my work more comfortable] (P017)	MAT-	17	11	I don’t have Internet access from home (P039)
SOC+	102	38	Help from a friend with shopping (P025)	SOC-	71	30	Communication with my manager and management was complicated (P022)
SCU+	52	23	The situation in Switzerland allowed us to go out every evening (P028)	SCU-	66	35	Working in the health field (…), the period was particularly busy professionally (P040)
TIME+	19	14	Having time to be together, accompanying them at school (P009)	TIME-	3	3	[I regret] not having had more time (P027)

*Note.* PEB, personal beliefs; COB, contextual beliefs; EMO, emotions; BIO, biological functioning; NAT, natural context; MAT, material context; SOC, social context; SCU, socio-cultural context.

### Incremental Value of the Three Types of Stories

**Figure 1.**
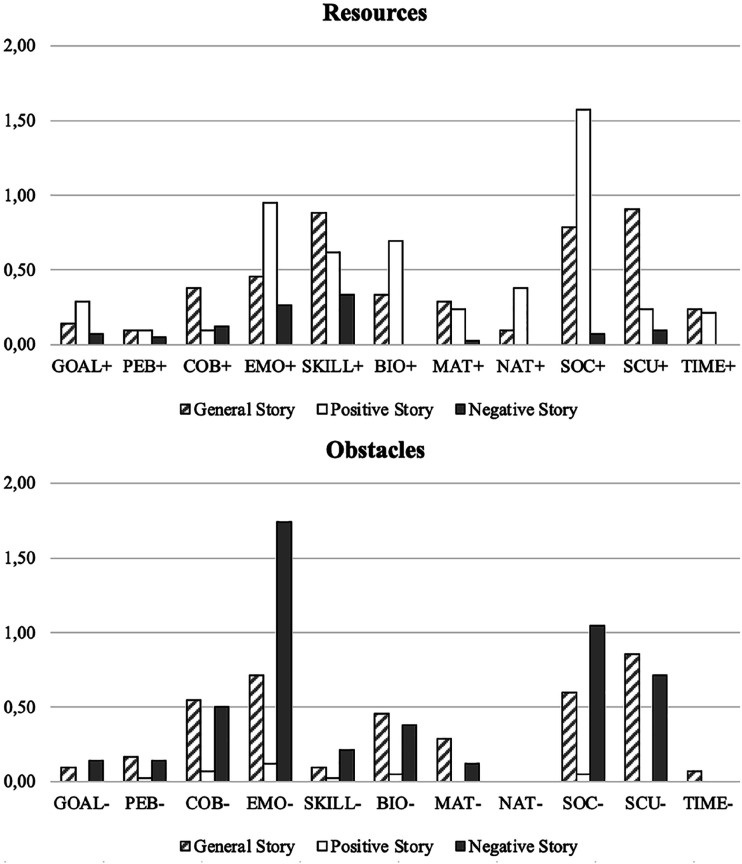
Mean number of resources and obstacle recounted per general, positive, and negative stories. *Note.* PEB, personal beliefs; COB, contextual beliefs; EMO, emotions; BIO, biological functioning; NAT, natural context; MAT, material context; SOC, social context; SCU, socio-cultural context.

Figure 1 displays the respective mention of total resources and obstacles mentioned within the three types of stories. Friedmans’ nonparametric ANOVA showed that the difference between the number of resources and obstacles mentioned significantly differed across the three types of stories. In fact, the General Story and the Positive Story contained significantly more resources than the Negative Story (χ^2^ = respectively 6.38, *p* < .001, and 7.98, *p* < .001). Similarly, participants tended to report fewer obstacles in the General and in the Positive stories than in the Negative Story (χ^2^ = respectively 2.26, *p* = .03, and 8.86, *p* < .001), as well as significantly fewer obstacles in the Positive Story versus in the General Story (χ^2^ = 6.61, *p* < .001; see Table 3). Additionally, the resources and obstacles mentioned within these three types of stories related differently to quantitative measures of well-being, perceived employability, and anticipation of the career future (see Table 4).

**Table 4.** Friedman’s Non-Parametric ANOVA at the Category Level.

	General Story		Positive Story		Negative Story		χ^2^	*df*	*p*
	M	SD	M	SD	M	SD			
Resources									
GOAL+	0.14	0.42	0.29	0.51	0.07	0.26	6.12	2.00	0.047
PEB+	0.10	0.30	0.10	0.30	0.05	0.31	1.52	2.00	0.468
COB+	0.38	0.70	0.10	0.30	0.12	0.40	10.10	2.00	0.006
EMO+	0.45	0.71	0.95	1.08	0.26	0.54	15.50	2.00	<.001
SKILL+	0.88	1.35	0.62	1.15	0.33	0.75	7.04	2.00	0.03
BIO+	0.33	0.57	0.69	0.98	0.00	0.00	24.10	2.00	<.001
MAT+	0.29	0.60	0.24	0.53	0.02	0.15	7.78	2.00	0.02
NAT+	0.10	0.37	0.38	0.82	0.00	0.00	10.10	2.00	0.006
SOC+	0.79	1.09	1.57	1.25	0.07	0.26	39.90	2.00	<.001
SCU+	0.90	1.30	0.24	0.62	0.10	0.43	19.80	2.00	<.001
TIME+	0.24	0.43	0.21	0.52	0.00	0.00	10.70	2.00	0.005
Obstacles									
GOAL-	0.10	0.48	0.00	0.00	0.14	0.52	5.14	2.00	0.076
PEB-	0.17	0.54	0.02	0.15	0.14	0.35	3.50	2.00	0.174
COB-	0.55	1.25	0.07	0.26	0.50	0.83	12.10	2.00	0.002
EMO-	0.71	1.11	0.12	0.33	1.74	1.34	39.60	2.00	<.001
SKILL-	0.10	0.37	0.02	0.15	0.21	0.52	5.09	2.00	0.078
BIO-	0.45	0.83	0.05	0.31	0.38	0.88	11.20	2.00	0.003
MAT-	0.29	0.67	0.00	0.00	0.12	0.33	9.24	2.00	0.01
NAT-	0.00	0.00	0.00	0.00	0.00	0.00	-	-	-
SOC-	0.60	0.91	0.05	0.22	1.05	0.99	29.40	2.00	<.001
SCU-	0.86	0.93	0.00	0.00	0.71	0.89	28.00	2.00	<.001
TIME-	0.07	0.26	0.00	0.00	0.00	0.00	6.00	2.00	0.05

*Note.* PEB, personal beliefs; COB, contextual beliefs; EMO, emotions; BIO, biological runctioning; NAT, natural context; MAT, material context; SOC, social context; SCU, socio-cultural context.

Spearman’s correlations showed that, in the General Story, resources were significantly associated with adaptive outcomes, Positive contextual beliefs (COB+) correlated positively, significantly, and strongly (.56) with positive emotional anticipation of the future career. Hierarchical regression showed that adding COB+ in the General Story (step 2) explained 21% more (adjusted *R*^2^ = .23) of the variance of the PANAS+ compared to COB+ in Positive and Negative Stories (step 1; adjusted *R*^2^ = .02), which is significant (F(1, 38) = 11.4, *p <* .01). Additionally, social (SOC+) and socio-cultural (SCU+) resources mentioned in the General Story were negatively and significantly (respectively −.34 and −.31) associated with negative emotional anticipation of the career future. However, hierarchical regressions indicated that the presence of these resources in the General Story (step 2) explained almost nothing (respectively 1% and 4%) more (adjusted *R*^2^_SOC_+ = −.03; adjusted *R*^2^_SCU_+ = .00) of the variance of the PANAS- (F_SOC_+(1, 38) = 0.56, *p.* = .46; F_SCU_+(1, 38) = 1.90, *p* = .18), compared to a first step comprising respectively SOC+ and SCU+ in Positive and Negative Stories (respectively adjusted *R*^2^ = −.02 and −.02).

On the contrary, in the Positive Story, only obstacles held significant relationships with the considered outcomes. In this type of story, the account of social obstacles (SOC-) was positively and significantly (.31) associated with positive expectations for work. However, hierarchical regression indicates that the mention of SOC- in the Positive Story (step 2) explained nothing (0%) more (adjusted *R*^2^ = 0.02) of the variance of the PANAS+ (F(1, 38) = 3.26, *p.* = .08), compared to SOC- in General and Negative Stories (step 1; adjusted *R*^2^ = .07). The mention of negative emotions (EMO-) in the Positive Story was significantly and negatively associated with both positive emotional expectations for work (−.41) and perceived employability (−.37). Hierarchical regressions show that adding EMO- in the Positive Story (step 2) explained 14% more (adjusted *R*^2^ = .09) of the variance of the PANAS+ compared to EMO- in General and Negative Stories (step 1; adjusted *R*^2^ = .03), which is significant (F(1, 38) = 6.39, *p <* .05). However, adding EMO- in the Positive story (step 2) explained 2% less of the variance of the perceived employability scale (step 2; adjusted *R*^2^ = .02) compared to EMO- in General and Negative Stories (step 1; adjusted *R*^2^ = .04), which was insignificant (F(1, 38) = 0.01; *p.* = .92).

In the Negative Story, the obstacles accounted for all the significant associations with the quantitative outcomes. In this type of story, the presence of negative contextual beliefs (COB-) was positively and significantly (.31) associated with negative emotional anticipation of career future. Nevertheless, hierarchical regression indicates that the mention of COB- in the Negative Story (step 2) explained almost nothing (2%) more (adjusted *R*^2^ = 0.17) of the variance of the PANAS- (F(1, 38) = 0.91, *p.* = .35), compared to COB- in General and Positive Stories (step 1; adjusted *R*^2^ = .11). Moreover, negative emotions (EMO-) correlated negatively and significantly with well-being (−.34). All the other associations were nonsignificant (see Table 5). However, hierarchical regression indicates that the mention of EMO- in the Negative Story (step 2) explained nothing (0%) more (adjusted *R*^2^ = 0.09) of the variance of the flourishing scale (F (1, 38) = 0.21, *p.* = .65), compared to EMO- in General and Positive Stories (step 1; adjusted *R*^2^ = .11).

**Table 5.** Details of Spearman’s Correlations Between the Number of Resources and Obstacles and the Scales per Type of Stories.

	General Story				Positive Story				Negative Story			
	FLOUR	EMPL	PANAS+	PANAS-	FLOUR	EMPL	PANAS+	PANAS-	FLOUR	EMPL	PANAS+	PANAS-
Resources												
GOAL+	−0.17	−0.06	−0.13	0.10	−0.08	0.07	0.11	0.03	−0.11	0.08	−0.21	0.05
PEB+	0.12	0.16	−0.09	−0.09	−0.24	−0.21	−0.09	0.02	0.13	0.16	0.01	−0.15
COB+	0.03	0.25	0.56***	−0.16	−0.26	−0.06	−0.09	−0.06	−0.12	−0.04	0.01	−0.30
EMO+	−0.23	0.07	0.16	0.10	−0.22	0.08	0.31*	−0.16	0.05	−0.06	−0.01	−0.11
SKILL+	−0.08	−0.03	0.19	−0.07	−0.18	−0.12	−0.09	−0.04	−0.22	−0.08	−0.08	0.09
BIO+	0.07	0.06	−0.01	−0.01	0.11	0.30	0.01	−0.13	-	-	-	-
NAT+	0.12	0.17	0.04	−0.14	0.05	0.20	0.03	−0.07	-	-	-	-
MAT+	0.18	−0.13	0.03	−0.09	−0.15	−0.13	0.05	0.00	0.06	−0.19	0.08	0.24
SOC+	0.17	0.15	0.21	−0.34*	−0.07	0.18	−0.05	0.14	−0.07	−0.05	−0.03	0.18
SCU+	−0.12	−0.04	0.08	−0.31*	−0.16	0.09	0.10	0.02	−0.14	−0.30	0.11	0.16
TIME+	0.01	−0.01	0.06	0.07	0.19	0.15	0.09	0.10	-	-	-	-
Obstacles												
GOAL-	0.14	0.28	0.06	0.12	-	-	-	-	0.14	0.25	0.01	0.12
PEB-	0.20	0.25	0.19	0.18	−0.19	−0.16	−0.21	0.23	−0.03	0.11	0.08	−0.23
COB-	−0.08	0.16	0.09	−0.09	0.00	0.10	0.04	0.20	0.05	0.24	−0.28	0.31*
EMO-	−0.07	0.19	0.02	0.25	0.01	−0.37*	−0.41**	0.09	−0.34*	0.00	−0.28	0.09
SKILL-	0.13	−0.06	−0.01	0.19	−0.25	−0.23	0.08	−0.02	0.05	0.21	−0.06	−0.10
BIO-	0.15	0.01	0.14	0.28	−0.15	−0.24	−0.19	−0.02	0.04	0.02	0.00	0.11
NAT-	-	-	-	-	-	-	-	-	·	·	·	·
MAT-	0.18	−0.10	0.00	0.09	-	-	-	-	0.15	0.18	0.10	−0.10
SOC-	0.04	−0.15	0.08	0.05	−0.08	0.01	0.31*	0.20	−0.14	0.05	0.09	0.03
SCU-	0.03	−0.19	−0.04	−0.05	-	-	-	-	0.05	0.22	−0.08	0.12
TIME-	−0.05	−0.02	−0.14	0.02	-	-	-	-	-	-	-	-

*Note.* *p < .05, **p < .01, ***p < .001.

FLOUR, flourishing scale; EMPL, perceived employability; PANAS+, positive emotional aticipation of future career; PANAS-, negative emotional anticipation of future career. PEB, personal beliefs; COB, contextual beliefs; EMO, emotions; BIO, biological functioning; NAT, natural context; MAT, material context; SOC, social context; SCU, socio-cultural context.

Accordingly, Hypothesis 2—which postulated an incremental value of the different type of stories—is only supported for the General and Positive Stories with regards to the positive emotional expectations for work.

## Discussion

### Conceptual Implications

The first contribution of this paper is to suggest M. E. Ford’s (1992) model of effective functioning as an exhaustive framework to assess resources in the context of career shock or transitions—including those that are not directly associated with career success, but do contribute nonetheless (see Steiner & Spurk, 2019). In doing so, we rose to the challenge described by Halbesleben et al. (2014) in suggesting a taxonomy of factors that can act as career resources or obstacles which is both concise and varied, broad and specific, relevant to various situations, distinguish the factor from its valence, and accounts for both gain and loss. Expanding prior literature, this model allows anchoring the selection of career resources in a sound integrative theoretical framework of individual behavior and development (i.e., the LSF; D. H. Ford, 1987), which also hold the potential of drawing meaningful connections with various fields of research (e.g., education, counseling and clinical psychology). The latest developments of the model (M. E. Ford & Smith, 2007, 2020) also provide interesting ventures to understand the dynamic relationships between these resources. Additionally, the qualitative empirical test of this model refined it by emphasizing *time* as an overlooked factor in both M. E. Ford’s (1992) model and previous frameworks for career resources, although time and temporality do have an important impact on career courses (Akkermans et al., 2024; Olry-Louis et al., 2022).

Second, this paper emphasizes the ambiguous nature of these concepts. In fact, in the recent developments of his model, M. E. Ford and Smith (2007, 2020) highlight the need for the factors to be *equipoised* to foster the effective functioning, thus acknowledging the fact that a factor can act both as a resource or an obstacle to goal pursuit given its strength and the circumstances. This consideration is well illustrated in our study given the fact that, with the notable exception of the natural context, all factors were mentioned both as resources and obstacles. Furthermore, our results indicate that the mention of social obstacles in the Positive Story was associated with positive anticipations for the career future, showing that, under certain circumstances, otherwise negative factors can have positive outcomes (Koert et al., 2011). In this case, it is likely that weakened social ties could lead to greater investment in the work sphere (e.g., Shockley & Singla, 2011). Additionally, our results showed that the type of story (i.e., General Story and Positive Story) in which some resources (i.e., positive contextual beliefs) and obstacles (i.e., negative emotions) differentially predict the positive emotional anticipations for the career future. This underscores how the very context in which factors are evoked is deeply relevant to understand their ultimate role as a career resource or an obstacle. In doing so, our research nuance relevance of prior research findings which assessed career resources and obstacles in a linear fashion (e.g., Brown et al., 2018).

These results tend to indicate that, while it is interesting to account for the whole set of resources and obstacles at a given time with M. E. Ford’s (1992) taxonomy, what is the most likely to impact how people feel about their career future ultimately relies on their cognitive and emotional perception of it (e.g., Lazarus & Folkman, 1984). In this study, marks of optimism when considering the whole situation appear as distinctive predictors of positive anticipations of the future, which is consistent with the great body of literature supporting, for example, the associations between optimism and effective emotional regulation strategies (Solberg Nes & Segerstrom, 2006) or career outcomes (Eva et al., 2020). Additionally, our results indicate that the mention of negative emotions when relating a past positive event could betray negative anticipations toward the career future. In fact, negative affective response to positive events (i.e., negative affect interference; Frewen et al., 2012) has been associated with depressive symptoms, which may impact the anticipations of the future (Jordan et al., 2021). Our results also support the mounting evidence of the importance of anticipatory emotions of career outcomes (Parmentier et al., 2021, 2022, 2023) in professional paths, as well as the relevance of developing capabilities to be able to appraise, express, regulate and use emotions to foster career development (see Pirsoul et al., 2023, for a meta-analysis).

### Methodological Implications

The third contribution of this study to the literature is that it provides a coding system that allows the rating of the different factors as resources or obstacles. In fact, this study is the first attempt to translate M. E. Ford’s (1992) model of effective functioning into an empirical coding system. The high inter-rater agreements tend to support the approachability and intuitive understanding of this coding system and categories. Furthermore, it appeared that the categories that were derived allow coding of almost all the statements included in the short stories, except for time-related resources or obstacles, as mentioned above. Note that the few neutral statements also pertained to M. E. Ford’s (1992) categories; they were just not phrased in a positive or negative way, which prevented from classifying them as resources or obstacles. The study added to the literature by demonstrating the relevance and the feasibility of adapting such a model into a coding system to account for the narrative of resources and obstacles following a career shock.

### Practical Implications

**Figure 2.**
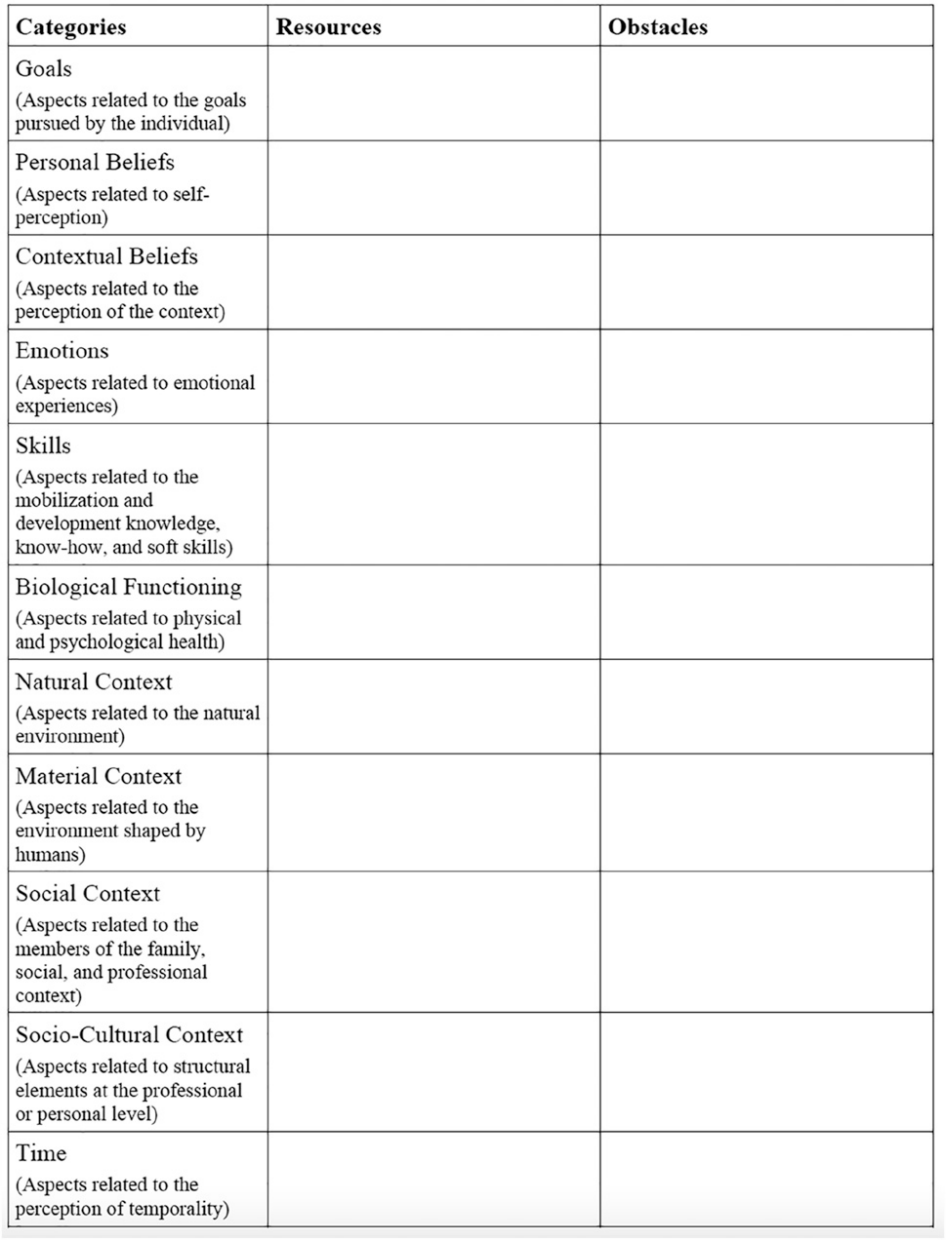
Note taking grid for qualitative interview by career practitioners.

Recently, several authors emphasized the need to map and foster individuals’ resources to assist with career transitions (Akkermans et al., 2024), career success (Hirschi, 2012; Hirschi et al., 2018), career sustainability (De Vos et al., 2020), and well-being at work (Donaldson & Donaldson, 2020; Donaldson et al., 2021). The major practical implication of this study is the demonstration that career resources (and obstacles) can be qualitatively assessed by listening to clients’ stories. In fact, the precent models focused on using quantitative instruments to assess career resources (e.g., PERMA+4 Short scale; Donaldson et al., 2023; Positive Functioning at Work Scale Donaldson & Donaldson, 2020; Career Resources Questionnaire; Hirschi et al., 2018; Marciniak et al., 2021). However, the resources and obstacles spontaneously evoked by people in their account of various life stories are revealing of their current well-being, as well as anticipation of their career future. Accordingly, career practitioners can draw inspiration from Bauer et al. (2019) narrative prompts to ask clients to provide them with a general account of their last few weeks, as well as to describe recent high and low points, in order to qualitatively identify some of their career resources and obstacles. In this effort, career practitioners would benefit from being familiarized with M. E. Ford’s (1992) categories to spot career resources and obstacles when listening to their clients. A note-taking grid could be used to record the different factors mentioned (see Figure 2).

Note that our results suggest that two types of factors are likely to act as influential resources or obstacles: positive contextual beliefs (in general stories) and negative emotions (in positive stories), which is akin to previous research results (e.g., Amundson & Borgen, 1987). Career practitioners could thus attempt to focus on the occurrence and valence of such factors in clients’ stories. Additionally, they can use card sorts and inventories that have been developed based on M. E. Ford’s (1992) model of effective functioning to foster the development and use of resources (RESILIENZA; Rochat, 2020) or identify the factors that led to a failure (DE’FEAT; Rochat, 2021). Finally, it should be noted that simple prompting of career clients to provide a narrative or their career shock or transition can have a beneficial impact on their well-being and adjustment, as stories offer ways to give meaning to career transitions (Olry-Louis & Arnoux-Nicolas, 2022) and to the events and foster the activation of resources (see Spera et al., 1994, for a study on expressive writing and coping with job loss).

### Limitations and Implications for Research

Our study has several limitations. First, the data were collected from a sample whose size is reasonable for qualitative research but limits the relevance of the quantitative findings. Accordingly, further studies will benefit to work on larger samples, which would allow to examine further the central role of positive contextual beliefs in general stories and of negative emotions on emotional anticipations of career path which was found in the present study. A larger sample would also allow to try and identify individuals’ distinctive profiles of career resources and well-being in response to a career shock or a transition (Butterfield et al., 2010). In fact, Hobfoll (2002) posits that resources tend to aggregate to form what he called *resources caravans*, implying that individuals will tend to be either high or low on resources in general. Person-centered (or within-person) approaches that test if individuals can be differentiated by their profiles (see Goodman et al., 2018) appears to hold promise as an effective way to try and identify individuals’ profiles based on the amount of resources (and obstacles) they display. Second, the sample consisted mostly of white women, and focused on full-time working individuals with permanent contracts who were working in the field of human and social sciences, and thus prevents us from accounting for the resources of marginalized and underserved populations (Blustein, 2006; Duffy et al., 2016). Accordingly, we encourage future studies to collect data among more diverse samples. A third limitation is that we did not ask the participants to take the Career Resources Questionnaire (Hirschi et al., 2018) nor Positive Functioning at Work Scale (Donaldson & Donaldson, 2020), which would have helped clarify the merits of both the qualitative and quantitative procedures and those two questionnaires to account for career resources. Further study may want to investigate these aspects in more detail.

## Conclusion

Relying on M. E. Ford’s (1992) model of effective functioning, the present study has emphasized the need for a broader and more complex view of resources in the context of career development. First, this study demonstrated that the categories of the model are relevant to account for almost all the resources and obstacles mentioned by the participants in their narratives of the lockdown experience—with the notable exception of time considerations, which appear as a relevant factor to add to the taxonomy. Second, our results show that the adaptative nature of the resources and obstacles mentioned differed according to the type of story narrated (i.e., general story, positive story, or negative story). This supports the claim that the potential role of certain factors to act as career resources versus obstacles should always be considered within the context of the specific experience. Finally, this study highlights the relevance and promises of having translated M. E. Ford’s (1992) taxonomy into an empirical coding system for both career practitioners and researchers.
